# Comment on: ‘Increases in arm volume predict lymphoedema and quality of life deficits after axillary surgery: a prospective cohort study.’

**DOI:** 10.1038/s41416-021-01267-3

**Published:** 2021-02-02

**Authors:** John Boyages, Chirag Shah, Frank A. Vicini

**Affiliations:** 1 Icon Cancer Centre, Wahroonga, NSW Australia; 2 Cleveland Clinic, Taussig Cancer Institute, Department of Radiation Oncology, Cleveland, OH USA; 3 21st Century Oncology, Michigan Healthcare Professionals, Farmington Hills, MI USA

**Keywords:** Breast cancer, Oedema

We commend Bundred et al. for completing their multicentre prospective study but, their claim that “bioimpedance spectroscopy (BIS) alone is not appropriate for lymphoedema screening or diagnosis”, does not fit with screening principles or current evidence, nor does their data support this conclusion.^1^

All patients underwent an axillary dissection and prescribed a compression garment when diagnosed with breast cancer-related lymphedema (BCRL), defined as perometry-based relative arm volume increase (RAVI) of >10%. This self-defined “gold-standard” was compared to other measures including bioimpedance spectroscopy (BIS) of >7.5 or ≥ 10 L-Dex units. BCRL (RAVI > 10%) was 22.4% at 24 months as compared with 45.2% for BIS ≥ 10 or 57.6% for BIS > 7.5.

We have several concerns with the study methods and conclusions. First, the study compares clinical lymphoedema (RAVI > 10%) to BIS, but the authors, in their introduction, noted a RAVI ≥ 5% as more suitable for early intervention. When this lower threshold was used, 51.4% met this criterion that closely correlated with BIS and symptoms **(**Fig. 1**)**. Early intervention is best implemented when there is subclinical BCRL (sBCRL) before adipose differentiation, and subsequent fibrosis occurs. Early detection of BIS changes and timely intervention with complex decongestive physiotherapy (CDP) is thought to reduce clinical progression.^2^ As clinicians treating breast cancer and contributing to BCRL, we have used BIS with over 35 years of collective experience and recognise its simplicity, reproducibility and patient acceptability.^3^ Waiting for clinical BCRL to develop, then prescribing a compression garment, is as inappropriate as waiting for breast cancer to become clinically palpable, rather than detecting it when not palpable with better outcomes.

**Fig. 1:**
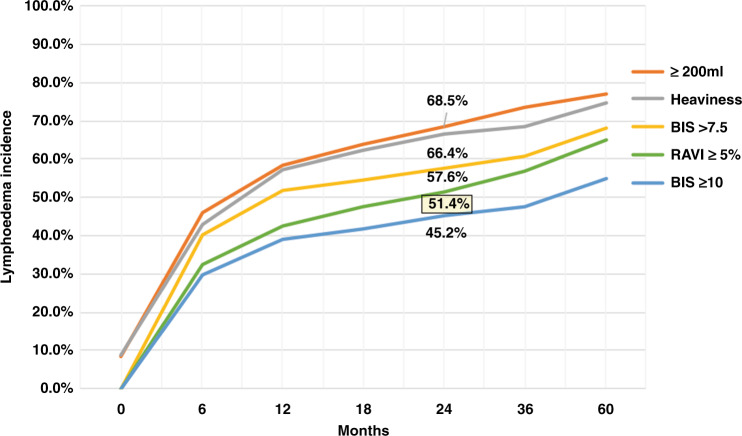
Probability of lymphoedema defined by the various volume or bioimpedance measures (derived from ref. ^1^). Highlighted percentage is the 24-month incidence of lymphoedema defined as a relative arm volume increase (RAVI) of ≥5%. BIS = bioimpedance spectroscopy (BIS) L-Dex units.

In contrast to the authors’ conclusions, previous studies have found that BIS is an objective measure for patients with sBCRL(>6.5). Early intervention with non-invasive measures (including skin care, self-massage and a 23–32 mm compression garment) are less intensive and less costly than CDP.^4^ BIS, particularly when performed using newer upright scale-like devices, provides immediate feedback and can be done quickly by any trained staff in several minutes.^5^ It has been used in Australia for 15-years and relatively easy to implement into clinical practice. Measuring girth when fat and fibrosis has set in is not the aim of L-Dex (or prospective surveillance) and not good clinical practice. The principle of early detection is to diagnose sBCRL which correlates with patients’ symptoms (suggesting an already developing pathologic condition) and, intervene with compression at that stage, which appears to assist reversal of the condition as demonstrated by ICG studies.^6^

A second concern is the study’s claim that a measure at 6-months predicts outcomes at 24 months, is difficult to justify when only 49.5% of patients were evaluable at 24 months, 28.5% at 36 months and 14.2% at 60 months which the authors attribute to the “anxiety-provoking” experience of coming to clinic. Our experience shows that regular BIS L-Dex checks can be done in radiotherapy, medical oncology, specimen collection laboratories or “lymphoedema departments” with excellent attendance rates^7^. We similarly do not stop checking follow-up mammograms based on this logic.

Finally, we are perplexed by the high rates of BIS increases and lower rates for RAVI. Ridner (2019) evaluated triggering rates for potential sBCRL by randomly comparing BIS (L-Dex ≥6.5) versus TM-derived RAVI > 5% and found that BIS had a lower rate (BIS:15.8% versus TM: 28.5%, *p* < 0.001) and longer times to trigger (BIS: 9.5 vs. TM 2.8 months, *p* = 0.002). BIS also reduced the absolute rates of progression of clinical BCRL requiring CDP by approximately 10% (TM: 14.7%, BIS: 4.9%), a clinically relevant improvement.^8^ This discrepancy may reflect the difference between perometry measures which can vary by arm position, the use of RAVI > 5% rather than 10%, the strict protocol conditions and the more complete follow-up in the Ridner study.^9^

Although the authors argue that the gold standard is RAVI ≥ 10%, we firmly believe that this is too late in the disease development for intervention to make a meaningful difference. The use of BIS together with TM as required is a cost-effective, patient-centred approach with the added advantage of measuring body composition so commonly affected by weight gain or chemotherapy-induced sarcopenia. Importantly, RAVI > 5% (i.e. subclinical BCRL) is highly correlated with L-Dex and the incidence at 24 months (51.4%), is midway between BIS ≥ 10 (45.2%) and BIS > 7.5 (57.6%) (Fig. 1).

L-Dex is convenient and less anxiety-provoking than perometry, which is a larger prohibitively expensive device, more efficient than TM-derived volume differences and can be implemented with information and psychological support to improve adherence to best-practice care.^10^

## Data Availability

Not applicable.
